# Dyskinesias after neural transplantation in Parkinson's disease: what do we know and what is next?

**DOI:** 10.1186/1741-7015-8-80

**Published:** 2010-12-02

**Authors:** Marios Politis

**Affiliations:** 1 Centre for Neuroscience, Division of Experimental Medicine, Faculty of Medicine and Neurology Group, Clinical Sciences Center, Medical Research Council, Hammersmith Hospital, Imperial College London, London W12 0NN, UK

## Abstract

Since the 1980 s, when cell transplantation into the brain as a cure for Parkinson's disease hit the headlines, several patients with Parkinson's disease have received transplantation of cells from aborted fetuses with the aim of replacing the dopamine cells destroyed by the disease. The results in human studies were unpredictable and raised controversy. Some patients showed remarkable improvement, but many of the patients who underwent transplantation experienced serious disabling adverse reactions, putting an end to human trials since the late 1990 s. These side effects consisted of patients' developing troublesome involuntary, uncontrolled movements in the absence of dopaminergic medication, so-called off-phase, graft-induced dyskinesias. Notwithstanding the several mechanisms having been proposed, the pathogenesis of this type of dyskinesias remained unclear and there was no effective treatment. It has been suggested that graft-induced dyskinesias could be related to fiber outgrowth from the graft causing increased dopamine release, that could be related to the failure of grafts to restore a precise distribution of dopaminergic synaptic contacts on host neurons or may also be induced by inflammatory and immune responses around the graft. A recent study, however, hypothesized that an important factor for the development of graft-induced dyskinesias could include the composition of the cell suspension and specifically that a high proportion of serotonergic neurons cografted in these transplants engage in nonphysiological properties such as false transmitter release. The findings from this study showed serotonergic hyperinnervation in the grafted striatum of two patients with Parkinson's disease who exhibited major motor recovery after transplantation with fetal mesencephalic tissue but later developed graft-induced dyskinesias. Moreover, the dyskinesias were significantly attenuated by administration of a serotonin agonist, which activates the inhibitory serotonin autoreceptors and attenuates transmitter release from serotonergic neurons, indicating that graft-induced dyskinesias were caused by the dense serotonergic innervation engaging in false transmitter release. Here the implications of the recent findings for the development of new human trials testing the safety and efficacy of cell transplantation in patients with Parkinson's disease are discussed.

## Introduction

Parkinson's disease (PD) is a common chronic neurodegenerative disorder characterized by the clinical presentation of motor (tremor, rigidity and bradykinesia) and nonmotor (e.g., autonomic, mood and cognitive) symptoms. Although the aetiology and pathogenetic mechanisms that cause PD remain unknown, classical descriptions of PD pathology mainly focus on the progressive degeneration of the nigrostriatal dopamine (DA) pathway and the pathology in other brainstem, cortical and subcortical structures [1]. PD patients are given DA replacement therapy for symptomatic relief, but these drugs prove beneficial up to a point and after a few years of L-3,4-dihydroxyphenylalanine (L-DOPA) therapy, the majority of PD patients develop motor complications, including abnormal involuntary movements called L-DOPA-induced dyskinesias (LIDs) [2]. As a result of this progressive decline in the clinical course of PD, more sophisticated therapeutic management has been warranted, one of which involves transplantation of fetal ventral mesencephalic (VM) tissue in the striatum of patients with PD. Human trials with fetal VM transplantation for PD have been conducted over the past two decades on the basis of the hypothesis that if PD is caused by degeneration of the nigrostriatal DA pathway and loss of DA innervation in the striatum, then restoration of the lost DA neurons by transplantation could reverse the loss of motor function. However, open-label trials and double-blind sham surgery controlled trials yielded inconsistent results and raised controversy [3]. Although some of the PD patients who underwent transplantation showed remarkable improvement of their motor symptoms, many of them had severe adverse reactions consisting of developing troublesome involuntary movements when off their DA drugs, called off-phase, graft-induced dyskinesias (GIDs) [4-6]. Whilst the exact mechanisms underlying the development of GIDs have remained unknown and there has been no effective treatment, proposed theories have been subject to extensive debate.

## Discussion

GIDs have been suggested to develop as a result of fiber outgrowth from the graft, causing increased DA release [4], or as the result of unevenly low or intermediate DA release from the striatum due to the imbalanced DA reinnervation [7]. However, two other studies argued against this view and reported no differences in either regional or global levels of striatal DA reinnervation between PD patients who have undergone transplantation with and without GIDs [6] and no correlation between GIDs and excessive DA reinnervation [5].

Other theories have included observations from animal models of PD and suggested that failure of the grafts to restore DA synaptic contacts with the host striatal neurons could result in abnormal signaling and abnormal synaptic plasticity in the transplanted striatum and therefore dyskinesia [8]. Furthermore, the occurrence of GIDs could be a result of inflammatory and immune responses around the graft. Clinical observations have suggested that GIDs develop after early discontinuation of immunosuppressive therapy [6,9], with signs of an inflammatory reaction around the grafts in autopsied cases [6]. An immunological reaction around the graft may cause a degree of tissue rejection, thereby diminishing the restoration of sustained striatal synaptic DA levels, which can be related to GIDs. Although both of these hypotheses appear plausible, they require experimental verification with animal and human studies.

Recent work from our lab [10] has focused on another population of neurons, namely, the serotonin (5-HT) neurons that appear in developmental stages caudally to the VM and were also transplanted. The hypothesis was based on the ability of 5-HT neurons to convert, store and release DA, as was shown after L-DOPA administration in animal models of PD [11]. Gain of function from a graft-derived, dense 5-HT terminal network in an otherwise restored striatal DA neuron function would lead to mishandling of the extracellular levels of striatal DA from the 5-HT neurons and would be responsible for the occurrence of GIDs.

Two patients with PD showing remarkable improvement of their motor symptoms more than a decade after striatal transplantation with fetal VM tissue, who received no DA drugs but developed disabling GIDs, were studied clinically and with positron emission tomography (PET) and radioligands tagging markers of presynaptic DA and 5-HT neurons. The results indicated excessive striatal 5-HT innervation in all grafted sites. Systemic administration of a 5-HT_1A _agonist, buspirone, which activates the inhibitory 5-HT autoreceptors and attenuates transmitter release from serotonergic neurons, resulted in significant attenuation of GIDs [10].

**Figure 1:**
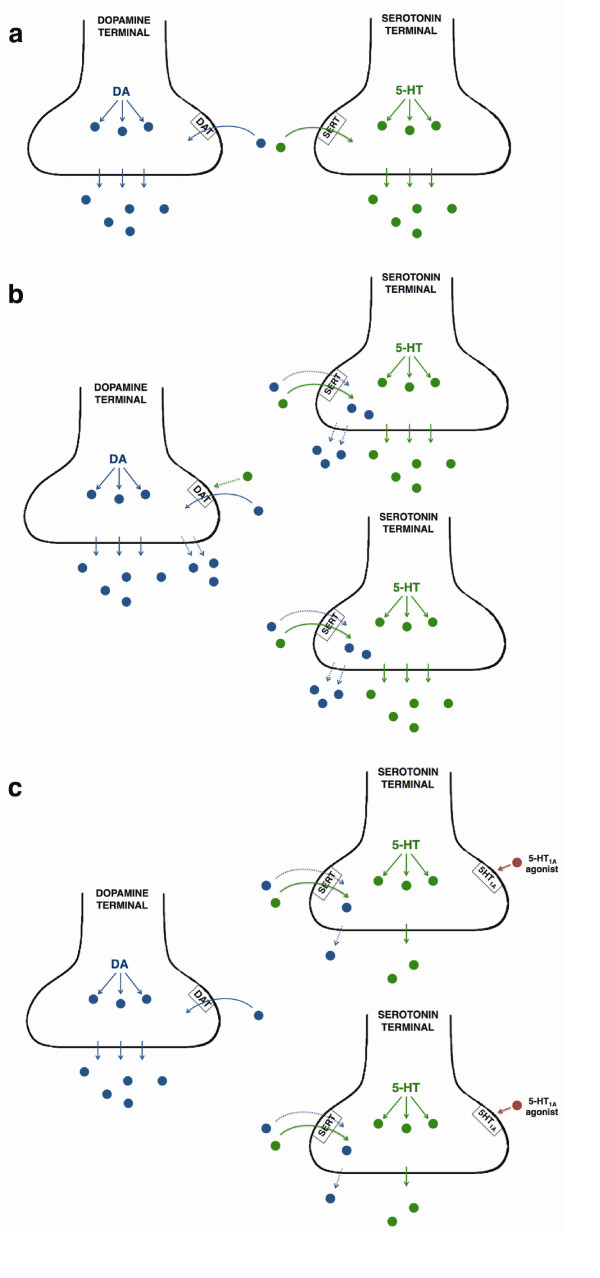
**Serotonin 1A (5-HT_1A_) agonists interfere with the 'dialog' between striatal dopamine (DA) and 5-HT neurons attenuating graft-induced dyskinesias (GIDs)**. **(a) **In healthy individuals or in patients with Parkinson's disease (PD) with successful striatal grafting and no GIDs, there is no dialog between DA and 5-HT neurons. The release of DA (blue dots) is regulated by presynaptic dopamine transporters (DAT) and the release of 5-HT (green dots) by serotonin transporters (SERT). **(b) **In patients with PD who have GIDs, the graft-derived striatal 5-HT hyperinnervation causes dysregulation in the synaptic levels of DA that lead to dyskinesias. 5-HT neurons can take up DA via SERT and release it as a false transmitter. Also, the excess of 5-HT release can act directly on DA neurons and reverse or alter the capacity of DAT, thus causing greater DA release from the grafted DA terminals. **(c) **Administration of 5-HT_1A _agonists (red dots) in patients with PD who have GIDs activate the inhibitory 5-HT autoreceptors, thereby diminishing the false release of DA and the excess release of 5-HT from the 5-HT terminals, leading to restoration of striatal synaptic levels of DA and attenuation of dyskinesias.

The results suggested that the occurrence of GIDs in these patients is caused by a 'dialog' between the graft-derived excess of 5-HT neurons and the normally functioning DA neurons (Figure 1). The hypothesis regarding this mechanism proposes that 5-HT neurons are responsible for dysregulating the DA release in the synapse. This could occur as a result of 5-HT transporters' taking up DA and releasing it as a 'false' transmitter from the 5-HT terminals and also as a result of the excessive 5-HT release acting directly in DA neurons and inducing further DA release by reversing the DA transporters (DAT). Administration of a 5-HT_1A _agonist (e.g., buspirone) interferes with this 'dialog' and by activating the inhibitory 5-HT autoreceptors attenuates the transmitter release from the 5-HT neurons, thus leaving most of the synaptic DA to be normally regulated from the DA terminals. The clinical correspondence of such interference is the significant attenuation of GIDs.

**Figure 2:**
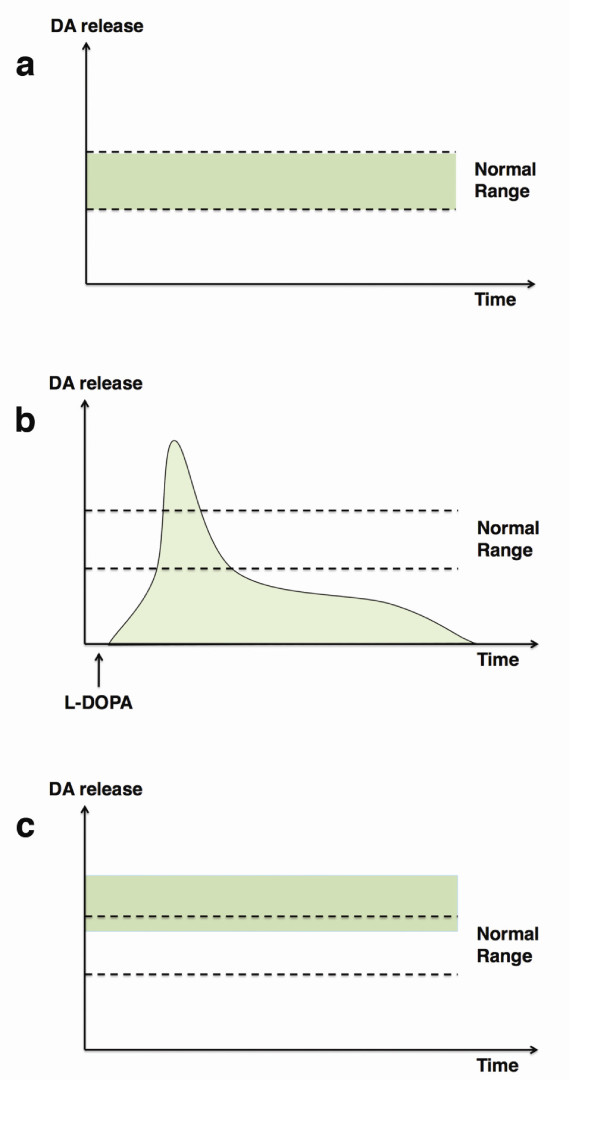
**Striatal DA release in peak-dose L-3,4-dihydroxyphenylalanine (L-DOPA)-induced dyskinesias (LIDs) and GIDs**. **(a) **Physiological release of DA in the striatum of healthy individuals. **(b) **Patients with PD who have peak-dose LIDs experience a sharp, short-term, abnormal increase in striatal synaptic DA concentrations when large doses of L-DOPA are administered and reach the extensively denervated striatum. Once the L-DOPA effect wears off, levels of DA drop below normal range. **(c) **Patients with PD who have striatal DA reinnervation rising to normal levels following transplantation with fetal ventral mesencephalic (VM) tissue experience constant GIDs because of the excessive 5-HT innervation in the grafted striatum. The 5-HT, neuron-derived, irregular levels in synaptic DA concentrations is a phenomenon with prolonged and steady duration and possibly lower intensity compared to LIDs.

Animal models of PD have recently demonstrated that removal of striatal 5-HT afferents or the dampening of 5-HT activity with 5-HT_1A _or 5-HT_1B _agonists resulted in attenuation of LIDs [12]. However, whether LIDs and GIDs share the exact same mechanism of DA dysregulation from 5-HT neurons is unclear. Patients with PD with peak-dose LIDs experience a radical and short-term increase in striatal synaptic DA levels when exogenous L-DOPA reaches the extensively denervated striatum [13]. In patients with PD who have restored DA innervation from transplantation but steady and constant presentation of GIDs, it seems plausible to assume that the 5-HT-driven abnormal release of DA is a phenomenon with prolonged and steady duration and possibly of lower intensity compared to the one observed in LIDs (Figure 2). This scenario is supported by observations of GIDs responding to systemic administration of low doses of 5-HT_1A _agonists [10], while LIDs responding to large bolus doses of the same drug aim for a more complete block of the concentrated 5-HT neuronal release of neurotransmitters [14].

## Conclusions

The occurrence of GIDs is a serious adverse reaction of striatal transplantation with fetal VM tissue in patients with PD hindering the development of future cell replacement therapies. The 5-HT hypothesis underlying the development of GIDs is the first hypothesis with experimental verification [10]. These recent findings have also supported a causative relationship between the striatal 5-HT hyperinnervation and the intensity of GIDs. According to these recent findings, GIDs could be prevented or treated. Patients with PD in its early stages lose about 15% and patients with advanced PD lose about 30%-35% of putaminal 5-HT neurons [15] compared to the profound DA denervation. Therefore, GIDs may be prevented by minimizing the serotonergic component in the grafted tissue during the dissection of VM, which should be very low in patients in the early stages of PD. Additionally, the use of fresh tissue should be applied because storage and culturing is expected to alter the proportion of cells in favor of non-DA cells such as 5-HT. GIDs could also be treated by the administration of 5-HT_1A _agonists, but the benefit could be greater and more practical by developing 5-HT_1A _agonists with sustained release.

However, more data are needed to fully establish the 'terms' of 5-HT/DA neuron 'dialog'. These data should come from transplanted PD patients with different degree of 5-HT/DA striatal reinnervation and should include those with incomplete DA reinnervation and those who do not experience GIDs.

The verification of the 5-HT hypothesis in the development of GIDs alongside the intense animal and human research of the past decade since the abandonment of fetal cell therapy in PD, indicates that we have come closer to designing new optimized protocols for the safer and more effective application of fetal cell transplantation in patients with PD.

## Abbreviations

DA: dopamine; DAT: dopamine transporter; GID: graft-induced dyskinesia; 5-HT: serotonin; LID: L-DOPA-induced dyskinesia; PD: Parkinson's disease; PET: positron emission tomography; SERT: serotonin transporters; VM: ventral mesencephalic.

## Competing interests

The authors declare that they have no competing interests.

## Authors' contributions

MP is entirely responsible for the content of this article.

## Pre-publication history

The pre-publication history for this paper can be accessed here:

http://www.biomedcentral.com/1741-7015/8/80/prepub
